# Synergistically Enhanced Photocatalytic Degradation by Coupling Slow-Photon Effect with Z-Scheme Charge Transfer in CdS QDs/IO-TiO_2_ Heterojunction

**DOI:** 10.3390/molecules28145437

**Published:** 2023-07-16

**Authors:** Li-Bang Zhu, Ning Bao, Qing Zhang, Shou-Nian Ding

**Affiliations:** 1 School of Chemistry and Chemical Engineering, Southeast University, Nanjing 211189, China; libangzhu@seu.edu.cn; 2 School of Public Health, Nantong University, Nantong 226019, China; ningbao@ntu.edu.cn; 3 Chinese Academy of Inspection and Quarantine, Beijing 100176, China; njuzhangqing@caiq.org.cn

**Keywords:** photocatalysis, slow photons effect, Z-scheme, inverse opal photonic crystal

## Abstract

Lower light absorption and faster carrier recombination are significant challenges in photocatalysis. This study introduces a novel approach to address these challenges by anchoring cadmium sulfide quantum dots (CdS QDs) on inverse opal (IO)-TiO_2_, which increases light absorption and promotes carriers’ separation by coupling slow-photon effect with Z-scheme charge transfer. Specifically, the IO-TiO_2_ was created by etching a polystyrene opal template, which resulted in a periodic structure that enhances light absorption by reflecting light in the stop band. The size of CdS quantum dots (QDs) was regulated to achieve appropriate alignment of energy bands between CdS QDs and IO-TiO_2_, promoting carrier transfer through alterations in charge transfer modes and resulting in synergistic-amplified photocatalysis. Theoretical simulations and electrochemical investigations demonstrated the coexistence of slow-photon effects and Z-scheme transfer. The system’s photodegradation performance was tested using rhodamine B as a model. This novel hierarchical structure of the Z-scheme heterojunction exhibits degradability 7.82 and 4.34 times greater than pristine CdS QDs and IO-TiO_2_, respectively. This study serves as a source of inspiration for enhancing the photocatalytic capabilities of IO-TiO_2_ and broadening its scope of potential applications.

## 1. Introduction

The rapid industrial development observed in recent decades has been accompanied by widespread environmental contamination, urgently requiring green and efficient solutions. Photocatalytic degradation, an emerging technology, offers a facile, controllable, and environmentally friendly approach when compared to traditional techniques such as adsorption, precipitation, and reverse osmosis [1,2]. This solar to chemical energy conversion is a process that harnesses sunlight and converts it into chemical energy, which can be stored and utilized for various applications. This conversion process involves the capture of solar energy through photovoltaic and its subsequent utilization to drive chemical reactions that store energy in the form of chemical bonds. Solar to chemical energy conversion plays a crucial role in achieving sustainable and renewable energy systems, as it allows for the efficient utilization of abundant and clean solar energy resources to produce storable and transportable chemical fuels. The above advantages are also highly attractive because it does not require additional energy [3,4,5], making it an increasingly popular choice in recent years [6,7,8].

The cornerstone of photocatalysis is photocatalytic material. In 1972, Fujishima et al. first reported TiO_2_ as electrodes for photocatalytic water splitting [6]. Since then, TiO_2_ has been intensively explored as a promising photocatalytic material due to its excellent chemical stability, durability, strong tenability, environmental compatibility, and cost effectiveness [9,10,11]. However, TiO_2_ has two notable limitations. Firstly, its large bandgap (3.2 eV) leads to lower light absorption, and secondly, it has a higher rate of photon-generated carrier recombination [12,13]. To address these limitations, researchers have explored various methods such as tailoring structures [14,15], doping non-metal heteroatoms [16,17,18], interacting with noble metal nanoparticles [19,20], and constructing heterostructures with narrower bandgap semiconductors [21,22,23]. Among these approaches, inverse opal (IO) photonic crystal, a periodic structure with unique optical and structural properties, has emerged as a promising solution due to its high light-harvesting efficiency [24,25,26,27]. The IO structure offers multiple benefits. Firstly, its open macroporous structure allows light to penetrate deeply into the photocatalyst [28,29]. Secondly, structural homogeneity enhances charge transport efficiency and reduces electron–hole recombination [30,31,32,33,34,35]. Additionally, the IO structure possesses a stopband due to its periodic refractive index, which prevents certain light from propagating in a particular crystallographic direction. At the “photonic bandgaps” (PBG) edge, the group velocity of photons becomes abnormally small due to nonlinear dispersion [36,37,38]. These slow photons have a longer lifetime and a larger propagation path length, which greatly enhances light absorption and improves conversion efficiency [39].

Polystyrene (PS) opal colloidal crystals were used as sacrificial templates to construct inverse opal TiO_2_ (IO-TiO_2_). However, the photocatalytic ability of the IO structure alone was insufficient, which led us to introduce a self-built-in electric field in IO-TiO_2_ to maximize its photocatalytic efficiency. In the context of photocatalysis, a self-built-in electric field refers to the generation of an electric field within a photocatalytic material without the need for external bias or applied voltage. This electric field arises as a result of the spatial distribution of charge carriers and the energy band alignment within the material. The self-built-in electric field plays a crucial role in enhancing the separation and migration of photo-generated charge carriers, namely electrons and holes. It creates a driving force that promotes the movement of these charge carriers toward their respective reaction sites, facilitating efficient redox reactions and minimizing recombination losses. The generation of a self-built-in electric field is often achieved through the design of heterojunctions or interfaces between different semiconducting materials. Generally, Type II heterojunction is a common and facile way to construct a self-built-in electric field [40,41,42]. It enhances spatially separating photogenerated electron–hole carriers through band alignment between two semiconductors [43]. Additionally, inside the type II heterojunction, the photogenerated electrons (or holes) could migrate from the higher potential to the lower one without any extra bias [44,45]. This distinguished performance makes it very suitable for biomaterials analysis. However, type II heterojunction also exhibits several problems when used as photocatalysts. The inherent electrostatic repulsion between the same charges makes it difficult for electrons or holes to migrate to the electron-rich conduction band (CB) or the hole-rich valence band (VB) [46,47]. Moreover, the lower reduction (oxidation) potential of type II heterojunctions seriously limits their redox ability in practical applications.

Inspired by plant photosynthesis, a new electron migration route exists in the chloroplast through photosystems I and II (PS I and PS II), which has perfectly solved defects of type II heterojunction. This electron migration route looks like the letter “Z”, thus denoted as the “Z-scheme” [48,49,50]. In the Z-scheme, unlike the electron migration in type-II heterojunction, photo-generated electrons can transfer from semiconductor A’s CB to semiconductor B’s VB (not from the CB to CB) due to the electrostatic attraction between the electron and hole [51,52]. This Z-scheme heterojunction not only preserves the efficient photo-generated carriers’ spatial separation but also retains the heterojunction with a higher redox potential, greatly enhancing its photocatalytic performance [53,54,55]. Semiconductor quantum dots (QDs) have recently gained attention in photocatalysis due to their unique properties, low cost, and effectiveness in light harvesting and charge separation [56,57,58]. In this undertaking, cadmium sulfide quantum dots (CdS QDs), a direct-band semi-conductor with a narrow bandgap, were selected and used to enhance the photocatalytic ability of IO-TiO_2_. The size of quantum dots is a critical factor that determines their properties. Generally, as the size of CdS QDs decreases, the bandgap energy increases, leading to a redshift in the absorption spectrum towards the visible light region. This allows CdS QDs to effectively utilize a broader range of solar irradiation for photocatalytic reactions. Veerathangam et al. significantly improved photoelectrochemical conversion efficiency by utilizing different-sized CdS QDs loaded onto the surface of titanium dioxide. Zhu et al. investigated the fluorescence effects of CdTe/CdS core–shell QDs with different sizes. Li et al. utilized CdS QDs with different sizes to enhance the photoelectrochemical conversion efficiency, which was applied in photoelectrochemical sensing [59,60,61]. Overall, the size-dependent properties of CdS QDs make them promising candidates for photocatalytic applications, and further research is being conducted to optimize their size and explore their full potential in various photocatalytic processes. By adjusting the reaction time, CdS QDs with various sizes (ranging from 2.7–4.7 nm) were prepared to obtain the optimal CdS QDs/IO-TiO_2_ heterojunction. To validate the photocatalytic performance of this heterojunction, we selected rhodamine B (RhB), a highly toxic heterocyclic dye with wide industrial application, as a model pollutant. The resulting photocatalysts successfully combined the slow-photon effect of the IO structure with the Z-scheme charge migration mechanism, resulting in improved photocatalytic performance. Compared with pristine CdS QDs and IO-TiO_2_, 4.7 nm of CdS QDs/IO-TiO_2_ photocatalyst exhibits promising photocatalysis degradation, and its photodegradable performance was 7.82 and 4.34 times higher, respectively. This study provides a constructive approach to optimize Z-scheme heterojunctions for efficient photocatalytic reactions.

## 2. Results and Discussion

### 2.1. Characterizations of As-Prepared IO-TiO_2_

**Figure 1:**
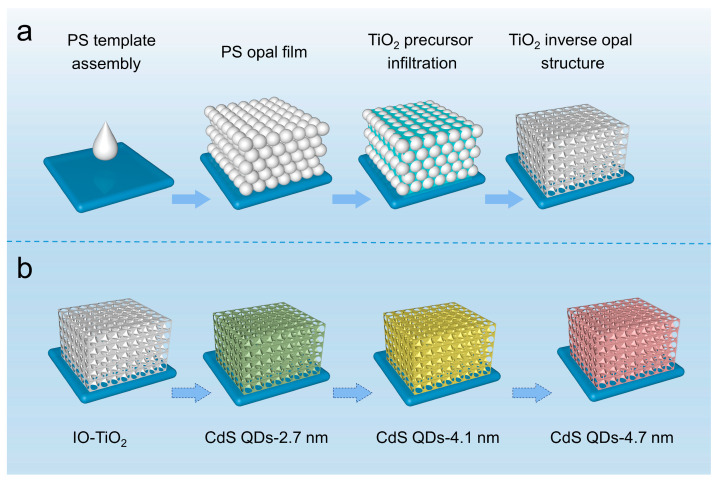
(**a**) The preparation process of IO-TiO_2_. (**b**) Various sizes of CdS QDs loaded with IO-TiO_2_.

**Figure 2:**
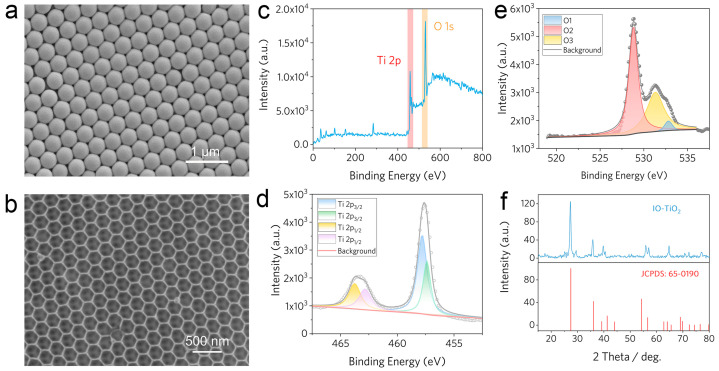
(**a**) SEM image of PS microspheres template, (**b**) prepared IO-TiO_2_, and (**c**) IO-TiO_2_ in micron scale. (**d**) XPS spectra of the IO-TiO_2_ and (**e**) Ti2p for IO-TiO_2_. (**f**) X-ray diffraction (XRD) pattern of IO-TiO_2_.

The preparation process of hierarchical IO-TiO_2_ is illustrated in Figure 1a, and the CdS QDs loaded IO-TiO_2_ are shown in Figure 1b. The detailed characterizations of IO-TiO_2_ are illustrated in Figure 2. The SEM image shows that the synthesized PS microspheres diameter is ca. 260 nm. In addition, the prepared PS colloidal crystal template is close-packed with a highly ordered, face-centered cubic arrangement (Figure 2a). Then, the TiO_2_ precursor penetrates the PS template, and the PS template is removed through calcination. Finally, a hierarchical IO-TiO_2_ structure was obtained, as presented in Figure 2b.

As shown, the as-obtained IO-TiO_2_ aperture is ca. 205 nm with a skeleton thickness of 25 nm. This interconnected macropore hierarchical structure could promote the fast transport of photo-generated carriers [62,63]. Compared with the corresponding PS templates, the pore sizes exhibit ~21% shrinkage, which is ineluctable in the calcination process. Nevertheless, it still formed highly ordered, crack-free micron scale inverse opal films (Figure S1). To confirm this material’s component, X-ray photoelectron spectroscopy (XPS) and X-ray diffraction (XRD) characterizations were carried out. XPS spectra show the existence of Ti2p and O1s (Figure 2c). Ti 2p_3/2_ and 2p_1/2_ of Ti^4+^ had corresponding binding energies of 457.9 eV and 463.7 eV, respectively (Figure 2d). The XPS spectrum of O 1s is three peaks (Figure 2e); this indicates three oxygen chemical states in the as-prepared sample. These results validated pure composition without chemical changes. The XRD pattern of prepared IO-TiO_2_ is highly analogous compared to the anatase TiO_2_ standard spectrum (JCPDS No: 65-0190) (Figure 2f).

### 2.2. Proof of the Slow-Photon Effect

The PBG wavelength (λ) of the inverse opal (IO) structure can be calculated following Bragg’s law Equation (1):

(1) $$\lambda =2\sqrt{\frac{2}{3}}D\sqrt{n_{{TiO}_{2}}^{2}f+n_{void}^{2}\left(1-f\right)-sin\theta }$$

where *D* is the IO-TiO_2_ aperture, n represents the refractive index, and *f* is the volume percentage of TiO_2_, commonly taken as 0.26. *θ* is the light incident angle. When the light incident angle is 0°, the wavelength (λ) is only related to the pore size (*D*).

**Figure 3:**
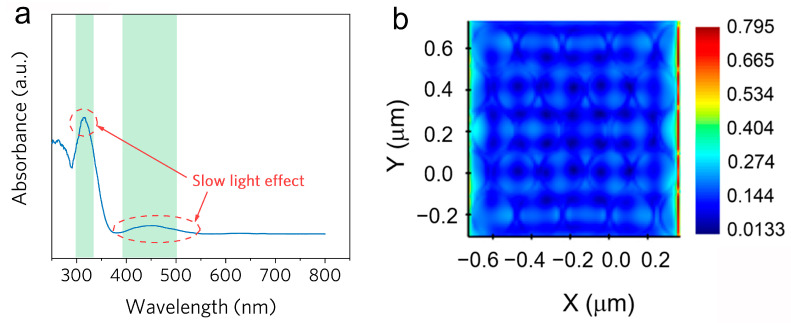
(**a**) UV–Vis DRS spectrum of prepared IO-TiO_2_. (**b**) Simulated photon transfer in IO-TiO_2_.

UV–Vis DRS shows various Bragg reflection peak positions of as-prepared IO-TiO_2_ centered at 477 and 322 nm (Figure 3a). To further prove the existence of PBG, we used Optiwave OptiFDTD 7 to carry out the theoretical simulation (Figure 3b). The finite-difference time-domain (FDTD) method was employed to calculate the electromagnetic field distribution by solving Maxwell’s equations. Figure 3b presents the simulated electromagnetic field intensity distribution. It is revealed that the enhanced electromagnetic fields are localized in the proximity of the IO-TiO_2_ interfaces [64].

### 2.3. Characterizations of Various Size CdS QDs

**Figure 4:**
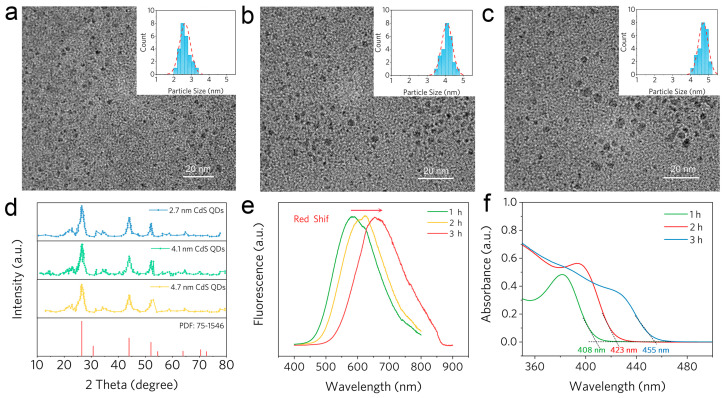
(**a**–**c**) TEM images and diameter statistics (inset) of various size CdS QDs. (**d**) X-ray diffraction (XRD) pattern of CdS QDs. (**e**) Fluorescence and (**f**) UV−Vis spectra of CdS QDs after different reaction times.

As shown, TEM images (Figure 4a–c) directly confirm the size of the CdS QDs. Depending on the heating duration, the average diameters of the prepared particles were 2.7 ± 0.2 nm (1 h), 4.1 ± 0.4 nm (2 h), and 4.7± 0.2 nm (3 h), respectively. The digital photograph of various size CdS QDs under ultraviolet light is also shown in Figure S2. The XRD pattern exhibited diffraction peaks positioned at 2θ = 26.5°, 43.9°, and 51.6°, which resembled the standard spectrum of cubic zinc blende form (JCPDS file 75-1546) (Figure 4d). The fluorescence spectrum (Figure 4e) also shows wide-ranging fluorescence peaks of CdS QDs, which are attributed to the semiconductor surface recombination of trapped charge carriers. Additionally, the quantum size effect causes fluorescence spectrum red shift. Furthermore, the increased CdS QDs diameter also causes the UV−Vis spectrum of CdS QDs (Figure 4f) to show well-defined absorption peaks at 408, 423, and 452 nm for 1 h, 2 h, and 3 h heating, respectively. As we know, the performance of QDs immensely depends on their size. The precise bandgaps of various size CdS QDs were calculated by Equation (2):

(2) Eg=1240λcut−off

After calculation, the precious bandgaps of various size CdS QDs are listed in Table 1. The corresponding bandgaps are 3.18 eV (408 nm), 3.05 eV (423 nm), and 2.82 eV (452 nm), respectively.

### 2.4. Characterizations of CdS QDs/IO-TiO_2_

**Figure 5:**
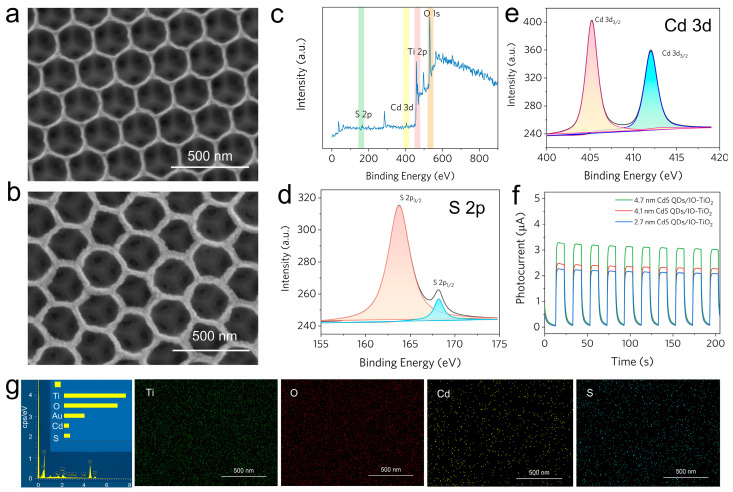
SEM image of (**a**) IO-TiO_2_, (**b**) CdS QDs /IO-TiO_2_, and (**c**) XPS spectra of CdS QDs/IO-TiO_2_. High-resolution XPS spectra of S 2p (**d**), Cd 3d (**e**). (**f**) Photocurrents of different size CdS QDs loaded onto IO-TiO_2_. (**g**) EDS element mapping of CdS QDs/IO-TiO_2_.

After coupling IO-TiO_2_ with CdS QDs, the smooth surface of IO-TiO_2_ became rough (Figure 5a,b). This indicates that the skeleton of the inverse opal structure provides abundant spatial sites for fixing CdS QDs. As illustrated in Figure 5c, the XPS survey spectra confirmed the coexistence of Ti (2p), Cd (3d), S (2p), and O (1s) in the CdS QDs/IO-TiO_2_ sample. The high-resolution XPS spectra (Figure 5d,e) established characteristic peaks at ca. 161 eV, 405 eV, and 412 eV, which represent S 2p, Cd 3d in CdS QDs. The effect of CdS QDs absorption time was investigated. Figure S3 shows that 180 min is the optimal absorption time. The photocurrent intensity (Figure 5f) of IO-TiO_2_ was enhanced with various CdS QDs in the visible light region. Figure 5g displays energy-dispersive X-ray spectroscopy (EDS) characterization. All peaks are well matched with the elements of CdS QDs/IO-TiO_2_. The homogenous fixing of CdS QDs on IO-TiO_2_ was clearly demonstrated by the element mapping analysis.

### 2.5. Band Structure of an Internal Electric Field

**Figure 6:**
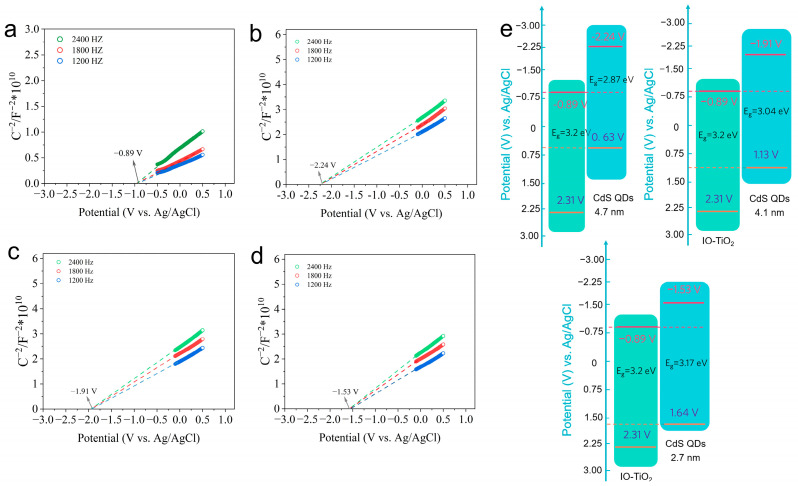
Mott−Schottky (M−S) plot of (**a**) IO-TiO_2_ and various size CdS QDs (**b**) 4.7 nm, (**c**) 4.1 nm, (**d**) 2.7 nm. (**e**) Band structure of IO-TiO_2_ and (i) 4.7 nm, (ii) 4.1 nm, and (iii) 2.7 nm CdS QDs.

A Mott–Schottky (M-S) plot of IO-TiO_2_ and various size CdS QDs are displayed in Figure 6a–d. Then, extending the M-S plots linearly, the intersections with abscissa (−0.89, −2.24, −191 and −1.53 V vs. Ag/AgCl) are the flat band (E_fb_) of the semiconductor. Furthermore, this positive slope of the M-S plot indicates their n-type semiconductor traits. As is well known, the n-type semiconductor conduction band potential (E_CB_) is approximately equal to E_fb_; thus, the E_CB_ of IO-TiO_2_ and various CdS QDs can be calculated as −0.89, −2.24, −1.91, and −1.53 V (vs. Ag/AgCl), respectively. Based on these calculated results, the energy band schematic illustrations of IO-TiO_2_ and various CdS QDs are elaborated in Figure 6e. The primary premise of constructing a Z-scheme heterojunction is matching band alignment; the CB of semiconductor A should be as near to semiconductor B’s VB as possible [65]. The appropriate VB (~0.63 eV) of 4.7 nm CdS QDs is the nearest to the IO-TiO_2_ CB, making it an optimal semiconductor to construct a Z-scheme heterojunction with IO-TiO_2_.

### 2.6. Photocatalytic Mechanism and Verification

**Figure 7:**
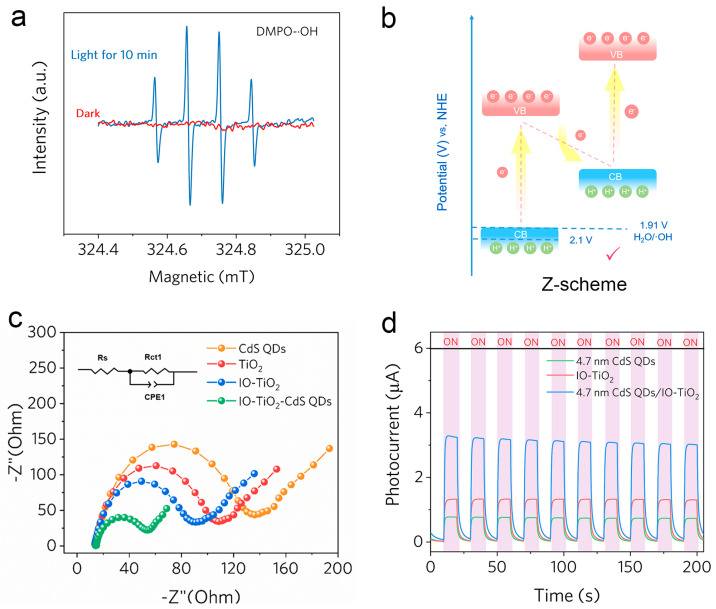
(**a**) EPR spectra of DMPO-∙OH. (**b**) Schematic illustration of Z-scheme charge migration pathway (**c**) EIS of CdS QDs, commercial TiO_2_ (P25), IO-TiO_2_, and (4.7 nm) CdS QDs/IO-TiO_2_ composites. (**d**) Photocurrent of 4.7 nm CdS QDs, IO-TiO_2_, and 4.7 nm CdS QDs/IO-TiO_2_ composites.

EPR spectroscopy was carried out to ascertain the generation of reactive oxygen species (ROS). In this experiment, DMPO was selected as a spin-trapping reagent, and a weak DMPO-∙OH signal can be observed in the dark. Upon photoexcitation, the characteristic peaks of DMPO-∙OH (1:2:2:1) can be monitored for CdS QDs/IO-TiO_2_ methanol dispersion liquid (Figure 7a). This result indicates that the charge migration route followed a standard Z-scheme transfer mode. Photoluminescence (PL) spectra are commonly employed to investigate the efficiency of charge carrier trapping, migration, and transfer in semiconductor particles. PL spectra provide valuable insights into the fate of electron–hole pairs as the PL emission originates from the recombination of free carriers. By analyzing PL spectra, researchers can gain a better understanding of the behavior and dynamics of charge carriers in semiconductors, which is crucial for optimizing the performance of various optoelectronic and photonic devices. The intensity of the PL emission spectra is determined by the recombination of excited electrons and holes. A lower PL emission intensity indicates a lower recombination rate in the samples, suggesting more favorable recombination properties. This can be attributed to efficient charge carrier trapping, migration, or transfer processes, which minimize the non-radiative recombination pathways and enhance overall photoluminescence efficiency. Thus, monitoring the PL emission intensity provides valuable information about the recombination behavior. Figure S4 shows the PL spectra of IO-TiO_2_, CdS QDs, and CdS QDs/IO-TiO_2_. The excitation wavelength was set at 300 nm to initiate the absorption of light by the sample. As shown, the IO-TiO_2_ exhibits strong emission peaks in the wavelength ranges of 380 to 430 nm. After the introduction of CdS QDs, a significant decrease in PL emission intensities was observed. In the heterostructures, the introduction of Au NPs and CdS nanolayers effectively suppressed the recombination of photogenerated electron–hole pairs. This can be observed by the decrease in photoluminescence (PL) emission intensities, indicating enhanced charge carrier trapping and reduced recombination processes. The presence of CdS QDs creates a favorable energy landscape for efficient charge transfer and separation, leading to improved photocatalytic performance. A schematic illustration of the detailed Z-scheme charge migration pathway in the heterojunction system is shown in Figure 7b. Under visible light irradiating, many photo-induced electrons (e^−^) with enough energy could jump into the CB of IO-TiO_2_ and CdS QDs, respectively. Then, the internal electric field could drive the electrons on the CB of IO-TiO_2_ to combine with the holes on the VB of CdS QDs, which could form the Z-scheme electron migration route. Finally, the photo-generated electrons aggregated on the CdS QDs’ CB and photo-generated holes accumulate on its VB. This electron transmission mechanism imparted superior redox ability to the Z-scheme system. Photocurrent measurement was also carried out to confirm the excellent photoelectric performance of CdS QDs/IO-TiO_2_. Figure 7c shows the electrochemical impedance spectroscopy (EIS) of pristine CdS QDS, IO-TiO_2_, and their composites. In comparison, CdS QDs/IO-TiO_2_ exhibits the smallest semicircle, while CdS QDs exhibit the largest. The semicircle of CdS QDs/IO-TiO_2_ is significantly decreased compared to that of pristine IO-TiO_2_ and CdS QDs, indicating the Z-scheme mode can reduce the interfacial charge transfer resistance. The photocurrent response provides the same conclusion. All the tested materials show a light response feature when exposed to a 300 W Xe lamp. (Figure 7d). The highest photocurrent observed for CdS QDs/IO-TiO_2_ is indicative of the most efficient separation and migration.

### 2.7. Photocatalytic Activity

**Figure 8:**
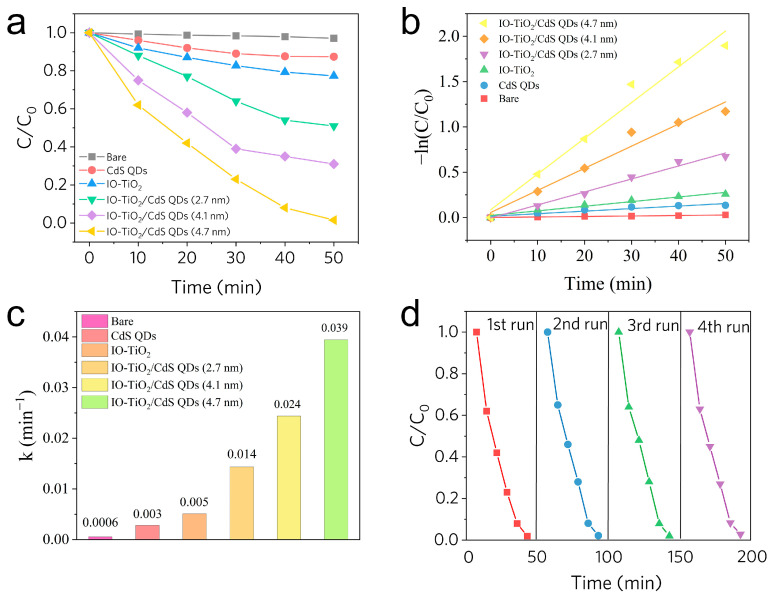
(**a**) RhB degradation results of samples. (**b**) Fitting diagram of the degradation rate under visible light irradiation. (**c**) The value of rate “k”. (**d**) Cycle tests of (4.7 nm) CdS QDs/IO−TiO_2_.

The investigation of CdS QDs/IO-TiO_2_ as a heterogeneous photocatalyst for RhB degradation was conducted. The corresponding results are presented in Figure 8a. As depicted, RhB exhibits minimal degradation when exposed to single-component CdS QDs and IO-TiO_2_ due to their poor charge separation. However, the degradation rate of CdS QDs/IO-TiO_2_ composites is notably improved, reaching up to 85% (4.7 nm CdS QDs/IO-TiO_2_, 50 min). The UV–Vis absorption spectra and digital photographs are shown in Figures S4 and S6. Additionally, the UV–Vis absorbance spectra of composites under dark adsorption are shown in Figure S5. These findings strongly support the Z-scheme migration mechanism contribution to the efficiency of the CdS QDs/IO-TiO_2_ heterojunction system. To better understand detailed photocatalytic degradation efficiency, the corresponding reaction rate constant “*k*” is calculated by kinetic fitting reaction degradation curve (Figure 8b). The calculation details follow Equation (3):

(3) $$-\ln\left(C/C_{0}\right)=kt$$

According to fitting results, the composite composed of 4.7 nm of CdS QDs demonstrates the highest degradation rate, being 13 and 7.8 times higher than that of pristine CdS QDs (0.003 min^−1^) and IO-TiO_2_ (0.005 min^−1^), respectively (Figure 8c). Moreover, as catalysts, it is essential to achieve high, stable catalytic activity. After the fourth recycling test, the prepared photocatalysts still maintained a high level of photocatalytic activity, indicating excellent reusability (Figure 8d). Additionally, the XRD pattern of the photocatalysts is shown in Figure S7, which reveals slight photo corrosion. It is worth noting that long-term exposure to light is inevitable.

## 3. Materials and Methods

### 3.1. Reagents and Apparatus

All reagents were purchased from commercial suppliers without further purification. Detailed information on the reagents and apparatus is available in Supplementary Materials.

### 3.2. Synthesis of PS Microspheres

PS spheres were synthesized according to the method in [54]. Firstly, 0.6 g potassium persulfate and 0.45 g sodium dodecyl sulfate were dissolved in 20 mL aqueous ethanol solution (50% *v*/*v*); then, the mixture was heated to 75 °C under a nitrogen atmosphere and 5 mL styrene was added. After continuous stirring for 19 h, the white emulsion (PS microspheres suspension) was obtained.

### 3.3. Assembly of Ordered PS Array Film Templates

The PS array film templates were assembled using the “evaporative deposition” method; a total of 500 μL of prepared PS sphere suspension was dispersed into 25 mL deionized water and sonicated for 30 min to ensure the dispersion was uniform. The cleaned ITO-coated glass slides (alternately, ultrasonically cleaned with ethanol, acetone, and deionized water for 5 min) were vertically immersed into the beaker and kept in a 75 °C oven. The hexagonal close-packed PS array film formed while the liquid was dried out.

### 3.4. Preparation of IO-TiO_2_ Structure

In this work, IO-TiO_2_ was prepared using an infiltration method. The prepared PS arrays film as sacrificial templates and are fabricated by filling the interstitial space between the spheres with the TiO_2_ precursor. The details are as follows: The TiO_2_ precursor, consisting of TiBALDH, 0.1M HCl, and ethanol (1:1:1.5 volume ratio), was stirred for 1 h at room temperature. Then, 10 μL precursor was deposited onto the as-prepared PS template and kept at 35 °C for 4 h. The electrodes were heated from room temperature to 450 °C at 1.0 °C·min^−1^ ramp rate and held for 2 h. Finally, the IO-TiO_2_ structure was formed.

### 3.5. Synthesis of CdS QDs

Synthesizing CdS QDs was based on a previous study with a slight, rutile modification [55]. Firstly, add 104 μL TGA (98%) to CdCl_2_·2.5H_2_O aqueous solution (34 mL, 0.175 mM) and thiourea (22 mg, 0.289 mmol). The molar ratio of Cd^2+^: thiourea: TGA was 1:1.7:2.3. Deaerate the mixture with nitrogen-bubbling for 30 min. Meanwhile, adjust the mixture to pH 10 with 1.0 M NaOH. Then, transfer into a Teflon-lined stainless steel autoclave, keeping the autoclave at 100 °C for a designed time and cooling to room temperature. Finally, purify the different sizes of CdS QDs three times using ethanol–water (*v*:*v* 1:4) and centrifugation at 10,000× *g* rpm for 5 min. The purified QDs were redispersed in water and stored at 4 °C for further use.

### 3.6. Preparation of CdS QDs/IO-TiO_2_ Heterojunction

The as-prepared IO-TiO_2_ ITO electrodes were immersed in the CdS QDs solution until they reached saturated adsorption. The whole process was carried out in a dry place, protected from light.

### 3.7. Measurement of Photocatalytic Activity

The CdS QDs/IO-TiO_2_ heterojunction photocatalytic effect was examined using the degrading organic contaminant RhB. In this experiment, CdS QDs/IO-TiO_2_ heterojunction photocatalyst (ca. 3 mg) was fixed on the ITO electrode and immersed in 30 mL RhB aqueous solution (ca. 50 mg·L^−1^). Then, this solution mixture was continuously agitated in the dark for 5 min to achieve saturation adsorption. A 300 W Xenon lamp (a cut-off wavelength of ~400–800 nm with 0.15 mW·cm^−2^ light intensity) recorded the photocatalytic responses. The mixture was sampled at certain intervals and then filtered through a 0.22 µm filter before UV spectrophotometer analysis. The digital photograph of the experimental setup is shown in Figure S8.

## 4. Conclusions

In this study, we have demonstrated the adaptability of the IO structure for further modifications such as the incorporation of quantum dots. By adjusting the size of CdS QDs loaded onto the IO-TiO_2_ surface, we achieved excellent synergistic photochemical amplification of the slow-photon effect and the Z-scheme charge transfer in CdS QDs/IO-TiO_2_. Herein, an extensive study has been conducted on the photoelectric properties, morphology, and structural characteristics of this material to better understand the enhanced mechanism. Thanks to this synergy, the novel hierarchical structure of the Z-scheme heterojunction exhibits degradability 7.82 and 4.34 times greater than pristine CdS QDs and IO-TiO_2_, respectively. This proof of concept allows for integrating various physical or chemical enhancements, making it valuable in photochemistry and related fields.

## Figures and Tables

**Table 1:** The λcut-off and bandgap (Eg) of different heating durations.

Heating Duration (h)	1	2	3
λ_cut-off_ (nm)	408	423	452
E_g_ (eV)	3.17	3.04	2.87

## Data Availability

Not applicable.

## Supplementary Materials

The following supporting information can be downloaded at: https://www.mdpi.com/article/10.3390/molecules28145437/s1, Reagents; Apparatus; Figure S1: SEM image of IO-TiO_2_ in micron scale; Figure S2: Digital photograph of various size CdS QDs under ultraviolet light; Figure S3: Effect of CdS QDs absorption time on the photocurrent responses of CdS QDs/IO-TiO_2_; Figure S4: UV–Vis spectra of RhB aqueous solution in the presence of (4.7 nm) CdS QDs/IO-TiO_2_ heterojunction under visible light irradiation; Figure S5: UV–Vis absorbance spectra of composites under dark adsorption; Figure S6: Digital photograph of RhB solution with increasing photocatalysis time; Figure S7: The XRD pattern of the composite before and after irradiation; Figure S8: Digital photograph of the experimental setup.

## Sample Availability

Samples of the CdS QDs/IO-TiO_2_ are available from the authors.
