# Supplementary material for: Synergistically Enhanced Photocatalytic Degradation by Coupling Slow-Photon Effect with Z-Scheme Charge Transfer in CdS QDs/IO-TiO2 Heterojunction
Source: Molecules. 2023 Jul 16;28(14):5437. doi: 10.3390/molecules28145437 (PMC10385498; doi:10.3390/molecules28145437)
Supplement: Supplementary file 1 [file molecules-28-05437-s001.zip › molecules-2495741-supplementary.pdf]

molecules

Supplementary material

Synergistically Enhanced Photocatalytic Degradation by Coupling Slow-Photon Effect with Z-Scheme Charge Transfer in CdS QDs/IO-TiO_2 Heterojunction

Li-Bang Zhu [1], Ning Bao [2], Qing Zhang [3], Shou-Nian Ding [1,*]

1. School of Chemistry and Chemical Engineering, Southeast University, Nanjing 211189, China; libangzhu@seu.edu.cn
2. School of Public Health, Nantong University, 226019 Nantong, Jiangsu, China; ningbao@ntu.edu.cn
3. Chinese Academy of Inspection and Quarantine, 100176 Beijing, China; njuzhangqing@caiq.org.cn

* Correspondence: snding@seu.edu.cn; Tel.: +86-25-52090621

Citation: To be added by editorial staff during production.

Academic Editor: Firstname Lastname

Received: date
Revised: date
Accepted: date
Published: date

Copyright: © 2023 by the authors. Submitted for possible open access publication under the terms and conditions of the Creative Commons Attribution (CC BY) license (https://creativecommons.org/licenses/by/4.0/).

1. **Reagents**

The indium tin oxide (ITO) slices (type N-STN-S1-10) were purchased from China Southern Glass Holding Co. (Shenzhen, China). Cadmium chloride ($CdCl_2$·2.5 H_2O, 99%) was purchased from Shanghai Jinshan Tingxin Chemical Reagent Co. Ltd. (Shanghai, China). Both 3-Mercaptopropionic acid (MPA, 99%) and thiourea (99%) were obtained from Sigma-Aldrich. Sodium hydroxide (NaOH), ammonium hydroxide (NH_3·H_2O, 25wt%), and anhydrous ethanol were purchased from Sinopharm Chemical Reagents Co. Ltd. (Shanghai, China). Dihydroxybis (ammonium lactate) titanium (IV) (TiBALDH) was purchased from Sinopharm Chemical Reagent Co., Ltd. (Shanghai, China). Potassium persulfate, sodium dodecyl sulfate, hydrogen chloride (HCl, 99%), and ethanol (99%) were obtained from Nanjing Chemical Reagent Co., Ltd. Styrene and rhodamine B (RhB) were purchased from Sigma-Aldrich. All materials were used without further purification. Ultrapure water (18.2 MΩ cm resistivity at 25 °C, Millier Q) was used in all experiments.

2. **Apparatus**

UV–visible absorption spectra were obtained on a UV-Vis-NIR-3600 spectrophotometer (Shimadzu, Japan). X-ray photoelectron spectroscopy (XPS) was performed on a PHI 5000 VersaProbe (UlVAC-PHI, Japan). XRD spectra were characterized by powder X-ray diffraction (XRD) [X'TRA, Cu Kα (ARL Co.)]. The morphology and microstructure of the PS template and IO-TiO_2 were recorded using a Hitachi S4800 scanning electron microscope (SEM) (Hitachi, Japan). A JEOL JEM-2100 transmission electron microscope (Hitachi, Japan) was employed to obtain high-resolution transmission electron microscopy (HRTEM) image of CdS QDs. Electrochemical impedance spectroscopy (EIS) was conducted with a CHI 760E electrochemical workstation (Chenhua Instrument, China). Electron paramagnetic resonance (EPR) was characterized with JEOL JES-FA200 (JEOL, Japan). The fluorescent spectra were performed on a FluoroMax-4 spectrofluorometer (Horiba, USA). All photocatalytic activities were measured under a light source 300W Xe lamp (40.25 Klux).

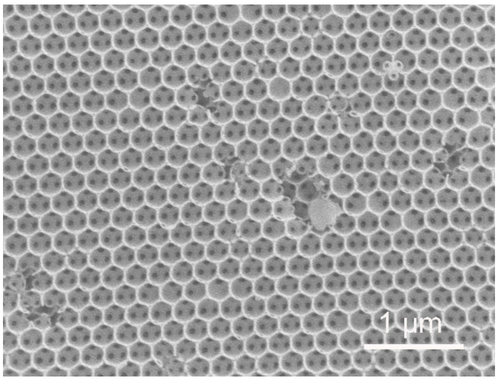

Figure S1. SEM image of IO-TiO_2 in micron scale.

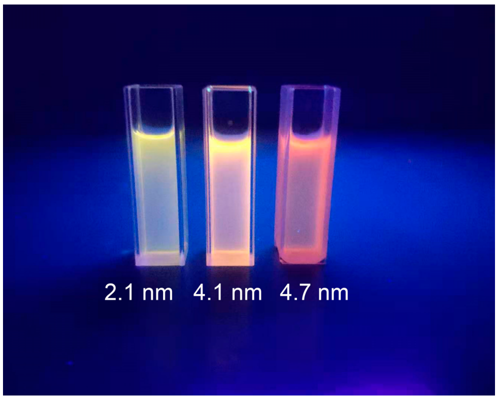

Figure S2. Digital photograph of various size CdS QDs under ultraviolet light.

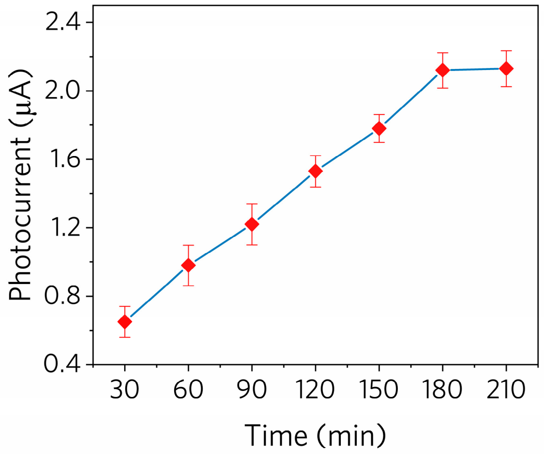

Figure S3. Effect of CdS QDs absorption time on the photocurrent responses of CdS QDs/IO-TiO_2.

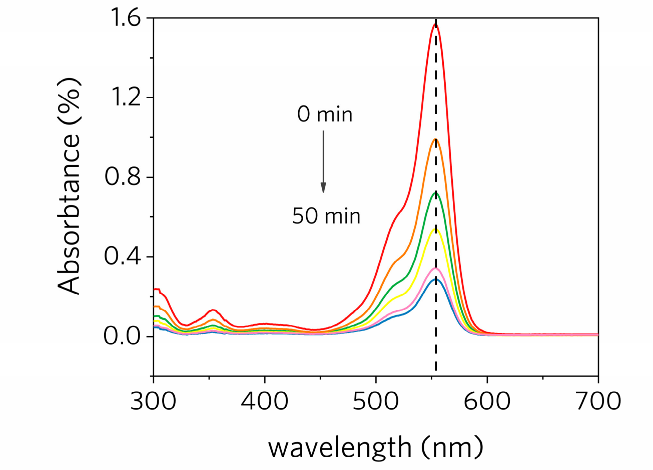

Figure S4. UV–Vis spectra of RhB aqueous solution in the presence of (4.7 nm) CdS QDs/IO-TiO_2 heterojunction under visible light irradiation.

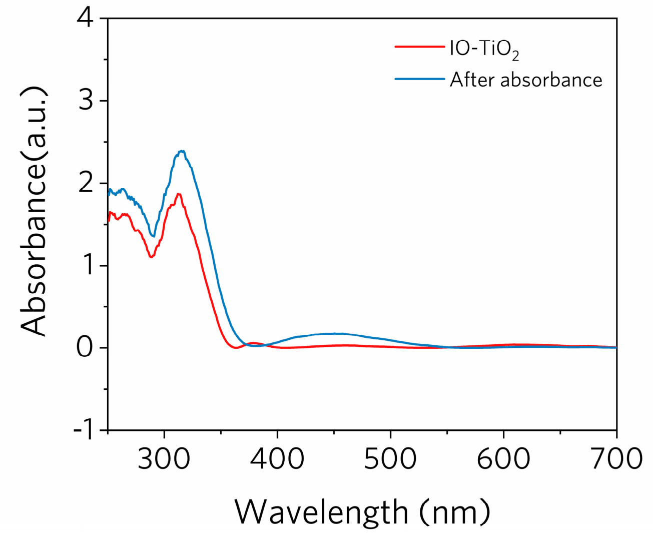

Figure S5. UV–Vis absorbance spectra of composites under dark adsorption.

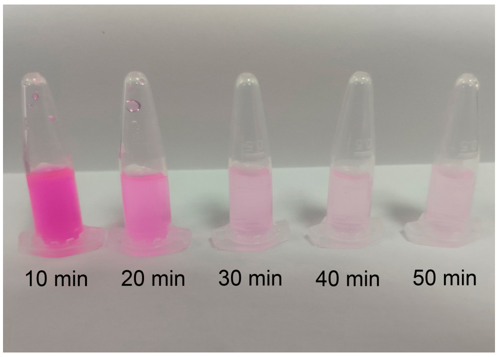

Figure S6. Digital photograph of RhB solution with increasing photocatalysis time.

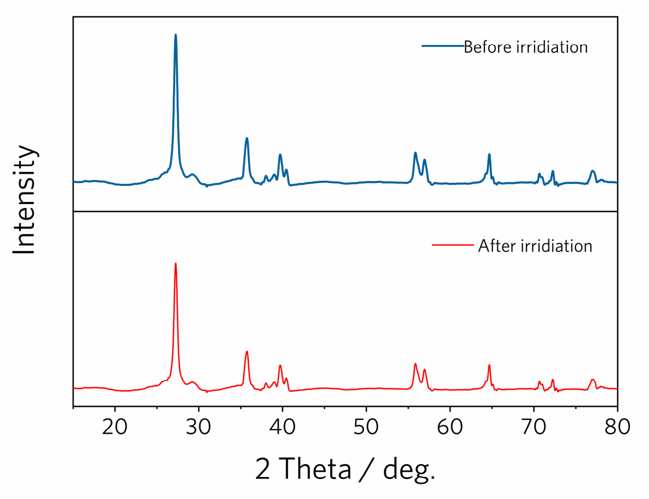

Figure S7. The XRD pattern of the composite before and after irradiation.

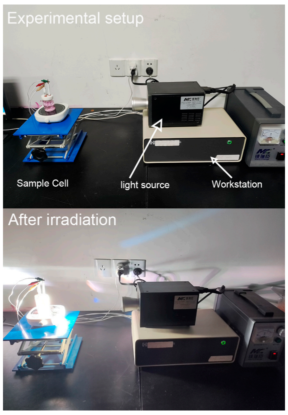

Figure S8. Digital photograph of the experimental setup.
